# Microstructure and Mechanical Properties of 34CrMo4 Steel for Gas Cylinders Formed by Hot Drawing and Flow Forming

**DOI:** 10.3390/ma12081351

**Published:** 2019-04-25

**Authors:** Yuebing Li, Wei Fang, Chuanyang Lu, Zengliang Gao, Xiakang Ma, Weiya Jin, Yufeng Ye, Fenghuai Wang

**Affiliations:** 1 Institute of Process Equipment and Control Engineering, Zhejiang University of Technology, Hangzhou 310032, China; ybli@zjut.edu.cn (Y.L.); 17857685226@163.com (W.F.); lvcykk@163.com (C.L.); jinweiya@zjut.edu.cn (W.J.); 2 Engineering Research Center of Process Equipment and Remanufacturing, Ministry of Education, Hangzhou 310032, China; 3 Zhejiang Jindun Pressure Vessel Co. Ltd., Shaoxing 312300, China; maxiakang@126.com; 4 Zhejiang Provincial Special Equipment Inspection and Research Institute, Hangzhou 310016, China; 13906536843@139.com (Y.Y.); wangfh@zjtj.org (F.W.); 5 Key Laboratory of Special Equipment Safety Testing Technology of Zhejiang Province, Hangzhou 310016, China

**Keywords:** flow forming, hot drawing, microstructure, mechanical properties, gas cylinder

## Abstract

An integral manufacturing process with hot drawing and cold flow forming was proposed for large-diameter seamless steel gas cylinders. The main purpose of this study was to find out the effects of the manufacturing process on the microstructure and mechanical properties of gas cylinders made of 34CrMo4 steel. Two preformed cylinders were produced by hot drawing. One cylinder was then further manufactured by cold flow forming. The experiments were carried out using three types of material sample, namely, base material (BM), hot drawing cylinder (HD), and cold flow-formed cylinder (CF). Tensile and impact tests were performed to examine the mechanical properties of the cylinders in longitudinal and transverse directions. Microstructure evolution was analyzed by scanning electron microscopy (SEM) and electron backscatter diffraction (EBSD) to reveal the relation between the mechanical properties and the microstructure of the material. It is found that the mechanical properties of the 34CrMo4 steel gas cylinders were significantly improved after hot drawing and flow forming plus a designed heat treatment, compared with the base material. The observations of microstructure features such as grain size, subgrain boundaries, and residual strain support the increase in mechanical properties due to the proposed manufacturing process.

## 1. Introduction

Gas cylinders are widely used to store and transport clean energy, such as compressed natural gas cylinders on vehicles and tube trailers for the transportation of hydrogen. These cylinders containing corrosive compressive gases are always operated under a high pressure over 20 MPa, which presents fatality risk and challenges for the safety of the gas cylinders throughout their anticipated long service life. Once the cylinders containing flammable gas rupture or leak, subsequent ignition is likely to occur, which can result in fire fatality [1,2]. Good performance characteristics of gas cylinders should not only ensure their safe operation, but also generate economic benefits. To optimize the performance of gas cylinders, especially for lightweight and long-life ones, several research studies have been dedicated to raw materials and manufacturing processes. A brief patent review of steel alloys used in the manufacture of gas cylinders was made by Nourani et al. [3]. Chromium-molybdenum steels with an excellent strength-to-weight ratio are currently used. One of the authorized steels for gas cylinders is 34CrMo4 (AISI 4130) steel which has superior corrosion resistance, mechanical properties, hardenability, and deformation characteristics.

To achieve an optimal structure for the cylinder end, the hot drawing process is widely used to fabricate gas cylinders [4,5]. The thermo-mechanical parameters of this process, such as temperature and strain rate, have a direct effect on the microstructure and mechanical properties of the cylinder material [3]. Rajan et al. [6] performed a study on the effect of heat treatment on the mechanical properties of flow-formed AISI 4130 steel tubes, by comparing the microstructures of the preform and finished flow-formed tubes using normalizing as well as hardening and tempering routes. The dynamic recrystallization characteristics of 34CrMo4 steel were investigated by hot compression tests at a temperature range of 900–1100 °C, a strain rate range of 0.001–0.1 s^−1^, and a strain of 0.9 [3,7]. For the mechanical properties of 4130 steel, hardening behaviors until high strain rates of 10^3^ s^−1^ and at elevated temperatures up to 1000 °C have been investigated by many researchers [8,9]. Recently, the stress–strain curves at various strain rates for 4130 steel were described with the modified Lim–Huh model, which includes the thermal softening effect [10] and the hyperbolic sine law in an Arrhenius-type equation [11].

In order to improve the performance and the final product quality of gas cylinders, the authors proposed an integral manufacturing process with a cold flow forming process for large-diameter seamless steel gas cylinders [5]. The flow forming process is most widely used to produce thin-walled, high-precision tubular products [12,13,14]. The effect of flow forming on material properties has also been studied with the evolution of microstructure and texture [15,16,17]. Rajan and Narasimhan [18] presented experimental observations of defects developed during flow forming of high-strength steel tubes and gave some advice for the flow forming parameters. Podder et al. [19] showed that variation in microstructural features and mechanical properties of the preforms due to heat treatments could significantly affect the flow formability and the deformation homogeneity of the resultant flow-formed tubes made of AISI 4340 steel. These research studies are usually limited to the laboratory test results. However, limited research has been carried out on the effect of cold flow forming on the material properties of the product cylinder. It is necessary to present the effects of the integral manufacturing process with cold flow forming process on the performance of gas cylinders using actual product materials.

This paper presents a systematic investigation of the effects of the manufacturing process proposed in [5] on the performance of gas cylinders, especially the effect of the cold flow forming on the product material properties. The mechanical properties of the gas cylinder materials before and after cold flow forming were tested and compared with the requirements of the material standard. The variation of the material with respect to microstructure, grain orientation, and boundary and local misorientation following the manufacturing process was also investigated.

## 2. Materials and Methods

### 2.1. Materials and Cylinders

**Figure 1:**
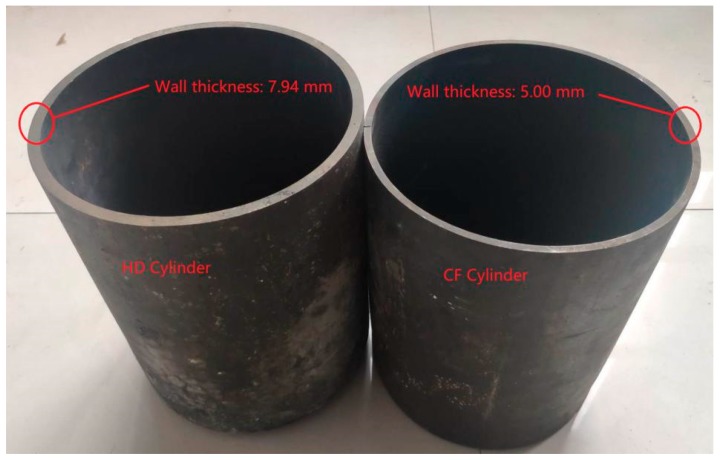
Gas cylinders with a diameter of 210 mm used in this work, including the hot drawing cylinder (HD) and the cold flow-formed cylinder (CF).

The billet used in this research was a block sample of 34CrMo4 steel. The chemical compositions of the base material were measured using an ARL 4460 optical emission spectrometer (Thermo Fisher Scientific, Waltham, MA, USA) and are given in Table 1, which satisfy the requirements of BS EN 10083-3: 2006 [20]. The base material is represented by “BM” in the rest of the paper. Two gas cylinders with an inner diameter of 210 mm (see Figure 1) were used to investigate the effect of the forming process on the material properties and the microstructures. The gas cylinder with a thickness of 7.94 mm was manufactured using the traditional process of hot drawing from the billet. This cylinder is referred to as the hot drawing cylinder and is represented by “HD” in the rest of the paper. Another cylinder was initially manufactured using the HD process and further fabricated by cold flow forming with a final thickness reduced to 5 mm. This cylinder is referred to as the cold flow-formed cylinder and is represented by “CF” in the rest of the paper. The CF cylinder was flow formed with a thickness reduction of 37% by a single-pass forming. The roller feed rate and mandrel rotation speed were controlled to be 1 mm/r and 180 r/min, respectively. In order to improve the toughness of the cylinders, a post heat treatment was introduced to both HD and CF cylinders after deformation. The cylinders were heated to 870 °C for 45 min and water quenched, and then tempered at 620 °C for 1.5 h followed by water cooling. To study the performance differences before and after the forming process, several tests for mechanical properties were carried out, including tensile tests, impact tests, and hardness tests. In addition, microstructure was observed using scanning electron microscopy (SEM) and electron backscatter diffraction (EBSD).

### 2.2. Tensile Tests

**Figure 2:**
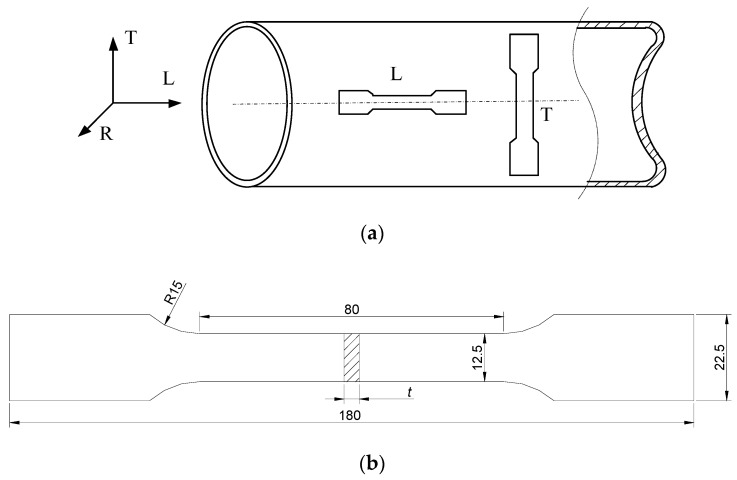
Specimens for tensile test, (**a**) Sampling direction (**b**) Specimen sizes.

Rectangular specimens for the tensile test, as shown in Figure 2a, were extracted from the HD and CF cylinders. A cylindrical coordinate system (L–R–T) was adopted to describe the sampling directions, where the L direction corresponds to the longitudinal or axial direction, the R direction corresponds to the radial direction, and the T direction corresponds to the transverse or circumferential direction. For both HD and CF cylinders, the tensile specimens for L and T directions were machined to compare the properties in longitudinal and transverse directions, respectively. The specimen sizes are shown in Figure 2b, where the thickness *t* is equal to the wall thickness of the cylinder. It should be noted that the T-direction specimens are nonplanar due to the curvature of the cylinders. Therefore, they were flattened mechanically before the test. The tensile tests were carried out using an Instron 8700 machine (Instron, Norwood, MA, USA) at ambient temperature under a loading rate of 0.2 mm/min.

### 2.3. Charpy Impact Tests

**Figure 3:**
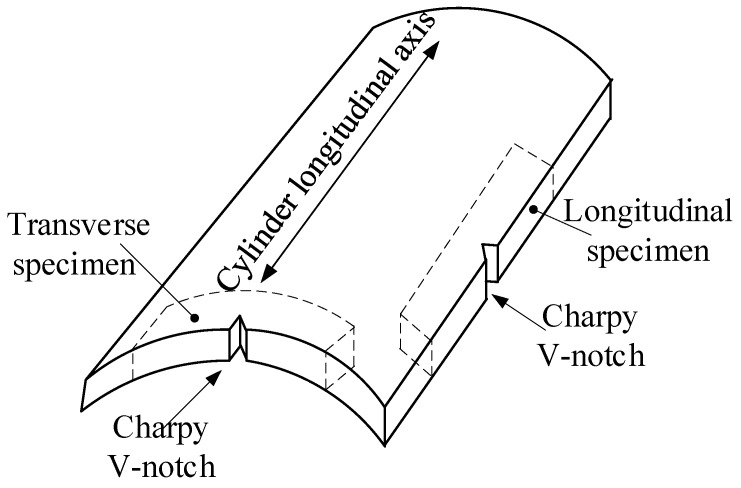
Specimens for impact test.

Similar to the tensile specimens, the impact test specimens were also extracted along L and T directions. For both L- and T-direction specimens, the through-wall thickness notch was machined (see Figure 3), and the specimen width is equal to the nominal wall-thickness of the cylinders. The impact tests were carried out on a ZBC-300A impact testing machine (MTS-SANS, Shanghai, China) at a temperature of −50 °C.

### 2.4. Hardness Tests

The hardness tests along the wall-thickness were carried out for both L- and T-direction specimens using the Vickers method, with a load of 0.05 kgf and 10 s loading time.

### 2.5. Microstructure Tests

**Figure 4:**
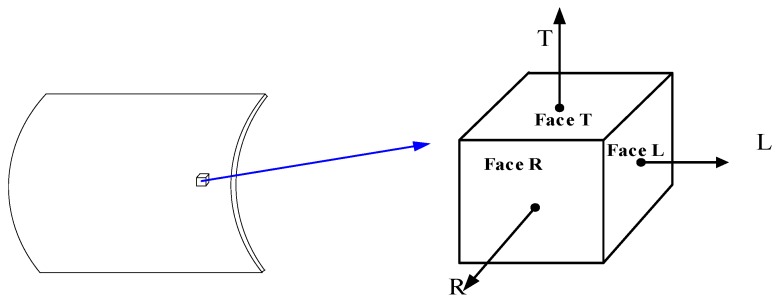
Specimens for microstructure and texture test.

Samples for microstructure tests were extracted from both HD and CF cylinders, as shown in Figure 4. Several samples were made with the face in the normal direction of L, R, and T, respectively. For each surface to be observed, it was initially grinded to 1500 mesh precision by sandpaper and polished to mirror surface on a polishing machine. The samples were then etched for 15 s in a solution consisting of 96% ethanol and 4% HNO_3_, then cleaned with alcohol. Optical microscope (OM) and SEM analysis were performed using a Nova Nano SEM450 type field emission scanning electron microscope (FEI Company, Hillsboro, OR, USA). Furthermore, after mechanical plus electrolytic polish of the L and R surfaces, an EBSD test was performed at a step size of 0.1 µm for both HD and CF samples, but 1 µm for the BM samples. The dimensions of the scan area were approximately 57 × 42 µm for the HD and CF samples, whereas they were 570 × 420 µm for the BM samples. The working distance was between 16 and 17 mm and the pixel binning used was 2 by 2. A scanning electron microscope equipped with a field emission type electron gun and a TSL/OIM EBSD system (EDAX Inc., Mahwah, NJ, USA) was used. The maximum accuracy of the EBSD scanning system was approximately 0.02 μm. For the EBSD scans, the sample was approximately 65–70° from the horizontal. The EBSD scan control, data acquisition, and orientation calibration were all carried out using the inbuilt software, TSL OIM Data Collection 5.0. Data such as average grain size and grain orientation difference angle of each view were recorded.

## 3. Results

### 3.1. Mechanical Properties

#### 3.1.1. Tensile Properties

**Figure 5:**
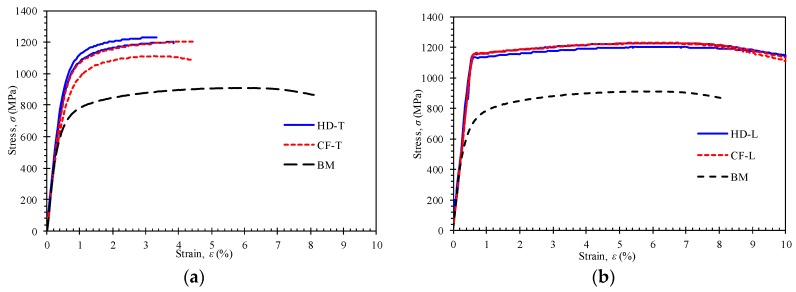
Stress–strain curve of 34CrMo4 for the (**a**) transverse (T) specimens and (**b**) longitudinal (L) specimens.

The basic mechanical properties of the materials were obtained via the tensile tests, including yield strength, ultimate tensile strength, and elongation. Figure 5 shows the stress–strain curves for the transverse specimens (Figure 5a) and longitudinal specimens (Figure 5b). The stress–strain curve for the BM specimens is also included in the figure. From Figure 5, the 34CrMo4 base material exhibits good ductility compared with the test results of the product materials. Compared with the base material, the tensile properties of the CF and HD materials are obviously enhanced. The tensile strength of the CF material is slightly lower compared with that of the HD material for the transverse specimens, while there is no obvious difference for the longitudinal specimens.

The measured mechanical properties for all specimens are given in Table 2. The results of the BM samples show that the strength and elongation satisfy the requirements of the standard [20]. The average yield strength and ultimate tensile strength of the CF and HD specimens are about 300 MPa higher than those of the base steel. The elongation of the HD and CF specimens is lower than the BM specimens but it is still higher than the standard requirement of 12%. It can be concluded that the mechanical properties of the product materials have changed after the manufacturing process and heat treatment compared with the base steel but that they still satisfy the requirements of the material standard.

For the average ultimate tensile strength of the formed components, small differences, namely, 16 MPa and −8 MPa in the longitudinal direction and transverse direction, respectively, are observed between the CF and HD specimens. For the average yield strength, the differences of 22 MPa and −13 MPa in longitudinal direction and transverse direction, respectively, are observed between the CF and HD specimens. It is concluded that the subsequent cold flow forming process does not cause significant changes in the mechanical properties of the material after heat treatment when compared with the results of the HD specimens.

It is found that the yield strength in the transverse direction is obviously lower than that in the longitudinal direction even in the same cylinder. The decrease is approximately 154 MPa and 189 MPa for the HD and CF specimens, respectively. In addition, there is a larger standard deviation in the transverse direction for both HD and CF specimens, compared with the longitudinal direction. This may be due to the amount of plastic deformation in the manufacturing process, which is different in longitudinal and transverse directions.

#### 3.1.2. Impact Properties

The impact test results for the two cylinders are shown in Table 3. It can be seen that the impact toughness of the CF specimens is higher than that of the hot drawing one, in both transverse and longitudinal directions. According to the results after cold flow forming, the average impact toughness increases about 23%. However, considering the difference in specimen size used in the tests for the two cylinders, this difference is insignificant. It can be concluded that the cold flow forming process does not reduce the impact toughness.

#### 3.1.3. Hardness

**Figure 6:**
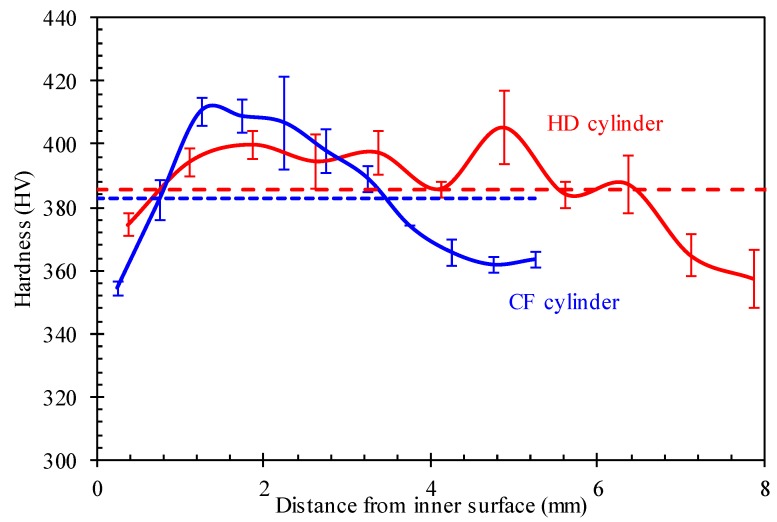
Measured hardness along the radial direction from the inner to the outer surface for the HD and CF cylinders.

Figure 6 shows the measured hardness along the radial direction from the inner to the outer surface for the HD and CF specimens. It can be seen from the figure that the average value of hardness in the whole thickness is very close for the HD and CF samples, as shown by the dashed line in Figure 6. The hardness values are uneven along the wall thickness, especially for the CF samples. The maximum hardness values are located in the mid-thickness region and the maximum deviations of the average values in the whole thickness are 48 HV and 56 HV for the HD and CF samples, respectively. It can be concluded that the cold flow forming process does not cause significant changes in hardness of the product material, although there is a difference in hardness distribution along the wall-thickness.

### 3.2. Microstructures

**Figure 7:**
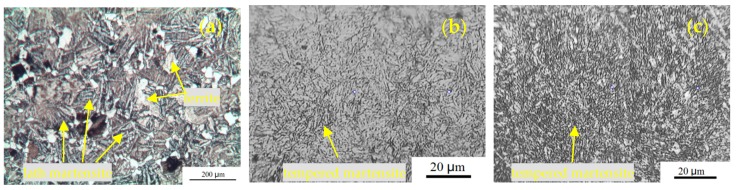
Optical microscope (OM) images for (**a**) base material (BM), (**b**) HD, and (**c**) CF materials.

**Figure 8:**
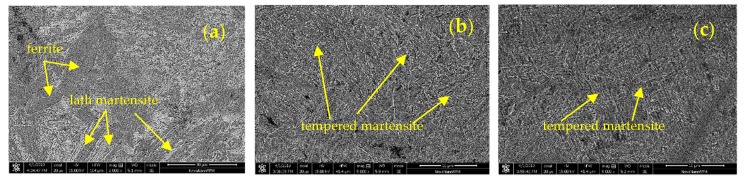
Scanning electron microscopy (SEM) images for (**a**) BM, (**b**) HD, and (**c**) CF materials.

Figure 7 shows the metallographic structures of the different samples. There is an obvious difference between the BM and the formed cylinder materials (HD and CF samples). For the BM, a large amount of lath martensite can be found with a small amount of ferrite. The microstructures are tempered martensite for the formed cylinder materials which have been heat treated by quenching and tempering. In addition, there is no significant difference in the metallographic structure between HD and CF materials. A similar result can be found by SEM, as shown in Figure 8. The microstructure of the BM is martensite and ferrite and that of the formed cylinder materials is tempered martensite.

**Figure 9:**
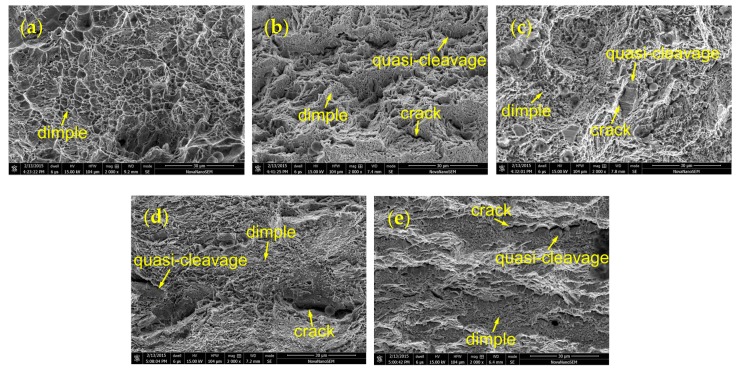
The fractography of tensile specimens for (**a**) BM, (**b**) HD-L, (**c**) HD-T, (**d**) CF-L, and (**e**) CF-T.

The fractography of tensile specimens for the different materials was analyzed using SEM, as shown in Figure 9. The fracture surface of the BM is basically characterized of dimples (see Figure 9a), indicating a ductile failure mode in the BM. The voids and cracks are found to be adjacent to the grain boundaries. The fracture surfaces of the HD and CF specimens are representative of quasi-cleavage characterized by the flat facets with tear ridges, and dimples, manifesting a mixed brittle/ductile failure mode, as shown in Figure 9b–e. At the same time, the serrated cracks and discontinuous voids are located at the edges of the flat facets. It can be inferred that the voids initially nucleate at grain boundaries and then grow and coalesce to become cracks along the grain boundaries, inducing a partial quasi-cleavage fracture in the HD and CF tensile specimens.

**Figure 10:**
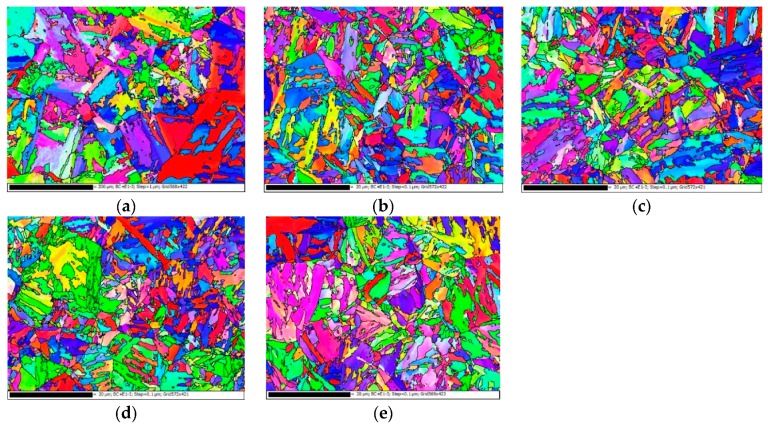
The orientation maps for (**a**) BM, (**b**) HD-L, (**c**) HD-T, (**d**) CF-L, and (**e**) CF-T.

Figure 10 shows the orientation maps for the BM as well as the HD and CF materials in both longitudinal and transverse directions obtained by EBSD. It can be observed that there are no distinct orientations of grains in these three materials and in both L and T directions. The average grain sizes of the BM, HD-L, HD-T, CF-L, and CF-T materials are 11.22, 1.02, 1.06, 1.00, and 1.07 μm, respectively. The grain size of the BM is approximately 11 times as large as those of the HD and CF materials in both longitudinal and transverse directions. The total numbers of grains investigated of the BM, HD-L, HD-T, CF-L, and CF-T materials are 917, 1331, 1280, 1540, and 1123, respectively.

**Figure 11:**
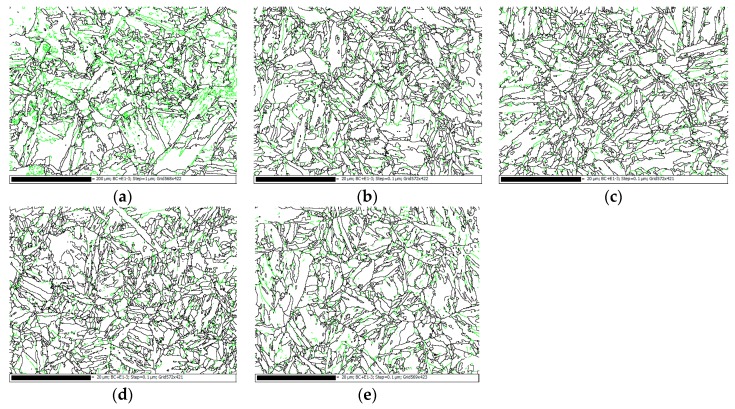
The grain boundary maps for (**a**) BM, (**b**) HD-L, (**c**) HD-T, (**d**) CF-L, and (**e**) CF-T.

**Figure 12:**
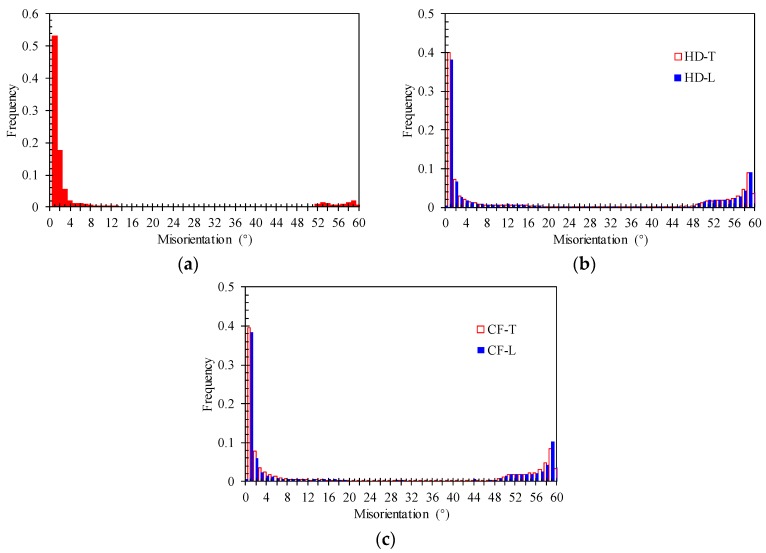
The misorientation distributions of grain boundaries for (**a**) BM, (**b**) HD, (**c**) CF.

Figure 11 illustrates the grain boundary maps for the BM, HD, and CF materials. In this figure, high-angle grain boundaries (HAGBs) with misorientation of more than 15° and low-angle grain boundaries (LAGBs) with misorientation of 5–15° are shown with black and green lines, respectively. The misorientation distributions of grain boundaries in different materials are shown in Figure 12. The initial microstructure shows a high percentage (~86%) of LAGBs, namely, the subgrain boundaries, in the BM, whereas the percentages of the HAGBs are relatively large (~40%) in both HD and CF materials.

**Figure 13:**
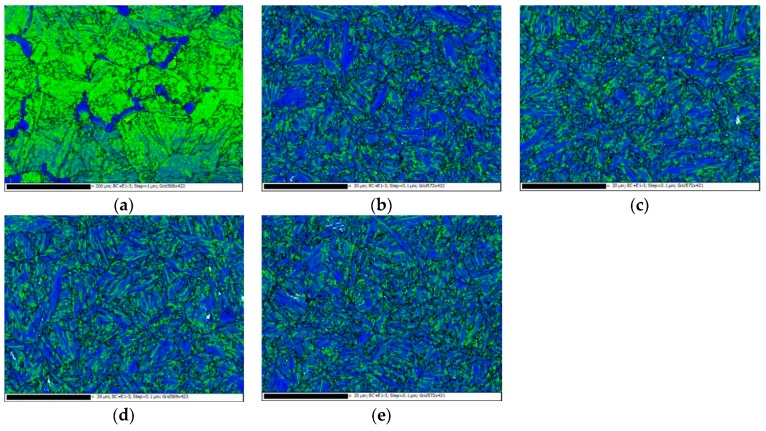
The local misorientation maps for (**a**) BM, (**b**) HD-L, (**c**) HD-T, (**d**) CF-L, and (**e**) CF-T.

The local misorientation maps corresponding to materials with different processes are shown in Figure 13. It is clear that the residual strain is obviously larger in the BM than that in the HD and CF materials. The concentrations of residual strain frequently occurred at the grain boundaries in the BM. There is still a certain amount of grains with little residual strain adjacent to the grains with high residual strain in the BM.

## 4. Discussion

The effect of sampling in tensile tests for the transverse specimens is first checked. Unlike the longitude samples, the flattening introduces a pre-strain on the transverse specimens. With reference to the cold-bending of steel plate to pipe [21], the strain *ε*(*x*) imparted due to flattening at a distance (*x*) from the mid-thickness of the thickness *t* and outer diameter *D* can be related by the following equation:

(1) $$\varepsilon \left(x\right)=\frac{2x}{D-t}.$$

The strain is negative from mid-thickness to outer surface and positive from mid-thickness to inner surface. The compressive strain at the outer surface is about 3.6% for the transverse specimen from the HD cylinder and 2.3% from the CF cylinder. During the flattening and the subsequent tension test, the outer wall is subjected to compression–tension strains, which indicates the Bauschinger effect. The Bauschinger effect results in a lower yield strength, which has been adopted to explain the decrease in yield strength of linepipe steels under repeated tension and compression strains [22,23]. Meanwhile, the process of flow forming brings about compressive strains in the cylinder transverse, which further reduces the yield strength due to the Bauschinger effect. Then, it drops more for CF than for HD. To sum up, the decrease in yield strength of the transverse specimens may result from the combination effect of flow forming and specimen preparation. Nevertheless, the strength properties of the cylinder exceed the minimum required high ultimate tensile strength (1200 MPa) and yield strength (900 MPa) [6].

From the microstructure view as shown in Figure 9, the non-distinct orientations of grains may result from the fact that the differences in tensile strength are not apparent in these three materials and in both L and T directions. HD and CF tested in the current work were heat treated after forming to eliminate the significant elongated grains along the longitudinal direction and residual stress. During the heat treatment of HD and CF, thermal recovery and recrystallization occurred, resulting in the fine equiaxed grains present in the HD and subsequent CF materials. In this way, the anisotropic characteristics in the HD and CF materials can be drastically alleviated, avoiding the extremely weak properties in certain orientations of the materials.

The slight differences in tensile strength can be deduced from the grain sizes. As mentioned above, the fine grains in the HD and CF materials were primarily caused by the heat treatment which induced the thermal recovery and recrystallization of materials. The grain sizes in the transverse direction are always larger than those in the longitudinal direction of both HD and CF materials, attributing to the extension of the longitudinal length in the HD and CF processes. Additionally, the grains of the HD material in the longitudinal direction are coarser than those of the CF material in the same direction, whereas it was the opposite in the transverse direction of the HD and CF materials. This phenomenon may be mainly attributed to the deformation mechanism of CF which reduced the cross-section area to extend the longitudinal length of the gas cylinder by external pressure in the radial direction. It should be noted that the volume of the gas cylinder was nearly constant during that process. Therefore, extruding the cylinder in the radial direction would stretch the grains along the longitudinal direction, leading to the growth of grain size in the transverse direction and the reduction of grain size in the longitudinal direction. It was therefore inferred that the CF process exerted an effect of refining grains in the longitudinal direction and coarsening grains in the transverse direction on CF materials. Meanwhile, the mechanical strength is considered to be predominantly dependent on the grain size according to the Hall–Petch relationship which can be expressed as follows [24]:

(2) $$\sigma_{s}=\sigma_{0}+kd^{0.5},$$

where *σ*_0_ is the material strength, σ_0_ is the intrinsic strength of a metal, *k* is the Hall–Petch constant, and *d* is the average grain size. Considering this, it is reasonable that the value of yield and ultimate tensile strengths are maximum in the CF-L materials and minimum in the BM because the grains are finest in the CF-L material and coarsest in the BM.

The impact property is also affected by the process of cold flow forming which results in the change of microstructure and crystallographic textures [16,25]. However, the gas cylinder after cold flow forming was heat treated. In consideration of the thermal recovery and recrystallization effects generated by the heat treatment, the dislocation density was therefore decreased, leading to the decline in the amount of subgrain boundaries appearing in the HD and CF materials. Although the ratio of subgrain boundaries was decreased in the HD and CF materials, the grain sizes of HD and CF were smaller than those of the BM, introducing a larger amount of grain boundaries per volume in the HD and CF materials than that in the BM. The grain boundaries are regarded as the barriers to the dislocation movements, which can produce a strain-hardening effect on the deformation and strength of the materials. The refined grains possess a higher resistance to brittle cleavage fracture since grain boundaries are effective barriers to the propagation of brittle fractures [26].

In general, the hardness affected by flow forming is reduced from the outer surface toward the inner layers [27,28]. It should be noted that the gas cylinder after cold flow forming was heat treated. Overall, this suggests that the appearance with respect to hardness is a combined effect of deformation and heat treatment. This trend was also found for high-pressure steel cylinders treated by quenching and tempering [29]. The explanation for that phenomenon may come from the samples’ microstructure [17]. The varied hardness indicates that the microstructures along the thickness are varied, which can be further investigated.

An attempt to reveal the performance characteristic of the gas cylinders was also made using the local misorientations. It has been stated that the local misorientation measured by EBSD can be a function of geometrically necessary dislocation (GND) density as expressed below [30]:

(3) $$\rho^{\mathrm{GND}}=\frac{2\theta }{\mu b},$$

where *ρ*^GND^ is the GND density at the investigated point, *θ* is the local misorientation angle, *µ* is the unit length of the point, and *b* is the Burger’s vector (~0.253 nm for steels [31]). Based on the relationship between local misorientation and GND density, it can be concluded that there were significant dispersions of dislocation density at the interfaces between high-residual-strain and low-residual-strain grains in the BM, resulting in the preferential stress concentration produced at these interfaces in the tensile and impact tests. It is therefore inferred that the voids and cracks would primarily initiate at the aforementioned interfaces, leading to the low yield and ultimate tensile strengths of the BM specimens. Due to the heat treatments after HD, thermal recovery was introduced to the HD material, dramatically reducing the dislocation density and residual strain in the material. The local misorientation, namely the residual strain, observed in Figure 13b,c is obviously lower in the HD materials than that in the BM. In the HD and CF materials, as displayed in Figure 13b–e, the residual strains are mainly aggregated at the subgrain boundaries, especially at the serrated boundaries and triple joints of grain boundaries. Consequently, the voids and cracks can be initially induced at and propagated along those sites during tensile and impact tests. This is in agreement with the inference from the fractography of the tensile specimens as shown in Figure 9. There is no obvious difference in the distributions of residual strain between the longitudinal and transverse directions of the HD and CF materials as shown in Figure 13b–e.

## 5. Conclusions

(1) The mechanical properties of the product cylinder materials all satisfy the requirements of the material standard. The tensile properties of the 34CrMo4 steel gas cylinders are obviously improved after the hot drawing and cold flow forming processes plus heat treatment compared with the base material. Therefore, the proposed manufacturing process and heat treatment used in the manufacture of the gas cylinders are acceptable.
(2) The mechanical properties and the impact toughness of the cold flow-formed cylinder are very similar to those of the hot drawing cylinder after heat treatment. Therefore, the cold forming process would not cause a reduction of the material’s strength and the process parameters and heat treatment plan are acceptable.
(3) The grain sizes of the HD and CF materials are significantly smaller than those of the BM, leading to the increase in the mechanical properties of the HD and CF materials. The microstructures of the HD and CF materials are very similar, including grain size, subgrain boundaries, and residual strain. This confirms the correctness of the processing parameters and the heat treatment plan used in the manufacture of the gas cylinders.
(4) The primary sites for voids and cracks during the mechanical tests of the BM are the interfaces with large dispersion of dislocation density. Meanwhile, the voids and cracks should be preferentially initiated at and propagated along the serrated boundaries and triple joints of the grain boundaries in the HD and CF materials during the tensile and impact tests.

## Figures and Tables

**Table 1:** Chemical composition of 34CrMo4 steel (%, mass fraction).

Source	C	Si	Mn	S	P	Cr	Mo	Ni	Al	Fe
Measured	0.36	0.23	0.71	0.003	0.012	1.06	0.23	0.044	0.022	Balance
BS EN 10083-3 [20]	0.30–0.37	Max. 0.40	0.60–0.90	Max. 0.035	Max. 0.025	0.90–1.20	0.15–0.30	-	-	-

**Table 2:** Tensile properties with average ± standard deviation.

Specimen Name	Yield Strength (MPa)	Ultimate Tensile Strength (MPa)	Elongation (%)
BM	674 ± 4.5	913 ± 3.1	16.0 ± 0.4
HD-L	1142 ± 10.3	1214 ± 11.4	16.0 ± 0.5
HD-T	988 ± 25.4	1215 ± 17	13.0 ± 0.1
CF-L	1164 ± 3.3	1230 ± 0.4	15.5 ± 0.5
CF-T	975 ± 23.7	1207 ± 0.2	12.5 ± 0.2
BS EN 10083-3 [20]	Min. 650	900–1100	12

**Table 3:** Impact properties with average ± standard deviation.

Specimen Name	Width (mm)	Depth (mm)	Cross-Section Area (cm^2^)	Impact Energy (J)	Impact Toughness (J/cm^2^)
HD-L	25	8.05	2.01	85.3 ± 5.3	42.4 ± 2.6
HD-T	25	8.10	2.03	85.5 ± 5.5	42.2 ± 2.7
CF-L	25	4.95	1.24	69.6 ± 4.5	54.3 ± 1.8
CF-T	25	5.75	1.44	75.0 ± 4.8	50.4 ± 1.7
